# Stuttering: Our Current Knowledge, Research Opportunities, and Ways to Address Critical Gaps

**DOI:** 10.1162/nol_a_00162

**Published:** 2025-04-02

**Authors:** Soo-Eun Chang, Jennifer E. Below, Ho Ming Chow, Frank H. Guenther, Amanda M. Hampton Wray, Eric S. Jackson, Ludo Max, Nicole E. Neef, Shahriar SheikhBahaei, Lana Shekim, Seth E. Tichenor, Bridget Walsh, Kate E. Watkins, J. Scott Yaruss, Nan Bernstein Ratner

**Affiliations:** Department of Psychiatry, Michigan Medicine, University of Michigan, Ann Arbor, MI, USA; Department of Communication Disorders, Ewha Womans University, Seoul, South Korea; The Vanderbilt Genetics Institute, Vanderbilt University Medical Center, Nashville, TN, USA; Department of Communication Sciences and Disorders, University of Delaware, Newark, DE, USA; Departments of Speech, Language, & Hearing Sciences and Biomedical Engineering, Boston University, Boston, MA, USA; Department of Communication Science and Disorders, University of Pittsburgh, Pittsburgh, PA, USA; Department of Communicative Sciences and Disorders, New York University, New York, NY, USA; Department of Speech & Hearing Sciences, University of Washington, Seattle, WA, USA; Department of Diagnostic and Interventional Neuroradiology, University Medical Center Göttingen, Georg August University, Göttingen, Germany; Neuron-Glia Signaling and Circuits Unit, National Institute of Neurological Disorders and Stroke (NINDS), National Institutes of Health (NIH), Bethesda, MD, USA; Department of Neurobiology and Behavior, Center for Nervous System Disorders, Stony Brook University, Stony Brook, NY, USA; National Institute on Deafness and other Communication Disorders (NIDCD), National Institutes of Health (NIH), Bethesda, MD, USA; Department of Speech-Language Pathology, Duquesne University, Pittsburgh, PA, USA; Department of Communicative Sciences and Disorders, Michigan State University, East Lansing, MI, USA; Department of Experimental Psychology, University of Oxford, Oxford, UK; Department of Hearing and Speech Sciences & Program in Neuroscience and Cognitive Science, University of Maryland, College Park, MD, USA

**Keywords:** genetics, intervention, neurobiology, research priorities, speech disorder, treatment

## Abstract

Our understanding of the neurobiological bases of stuttering remains limited, hampering development of effective treatments that are informed by basic science. Stuttering affects more than 5% of all preschool-age children and remains chronic in approximately 1% of adults worldwide. As a condition that affects a most fundamental human ability to engage in fluid and spontaneous verbal communication, stuttering can have substantial psychosocial, occupational, and educational impacts on those who are affected. This article summarizes invited talks and breakout sessions that were held in June 2023 as part of a 2-day workshop sponsored by the US National Institute on Deafness and Other Communication Disorders. The workshop encompassed topics including neurobiology, genetics, speech motor control, cognitive, social, and emotional impacts, and intervention. Updates on current research in these areas were summarized by each speaker, and critical gaps and priorities for future research were raised, and then discussed by participants. Research talks were followed by smaller, moderated breakout sessions intended to elicit diverse perspectives, including on the matter of defining therapeutic targets for stuttering. A major concern that emerged following participant discussion was whether priorities for treatment in older children and adults should focus on targeting core speech symptoms of stuttering, or on embracing effective communication regardless of whether the speaker exhibits overt stuttering. This article concludes with accumulated convergent points endorsed by most attendees on research and clinical priorities that may lead to breakthroughs with substantial potential to contribute to bettering the lives of those living with this complex speech disorder.

## INTRODUCTION

Stuttering is a childhood-onset neurodevelopmental disorder that involves disruptions in speech production. The outward manifestation of involuntary disruptions in the forward flow of speech is experienced by the speaker as loss of control, often accompanied by struggle, compensatory behaviors, and a generally negative impact on life. The severity of symptoms is highly variable, and stuttering is commonly exacerbated and influenced by a wide array of social, emotional, and linguistic contexts. As a condition that affects the fundamental human ability for verbal communication, stuttering can lead to negative occupational and psychosocial consequences throughout one’s life. Despite a relatively high life-time incidence and strong demand for informed treatment, our understanding of the biological bases of stuttering remains limited. Current treatment approaches often lead to inadequate, mixed outcomes, and few randomized controlled trials have been conducted to evaluate current or proposed treatment approaches for either children or adults who stutter (Brignell et al., 2020; Laiho et al., 2022).

**Figure 1.**
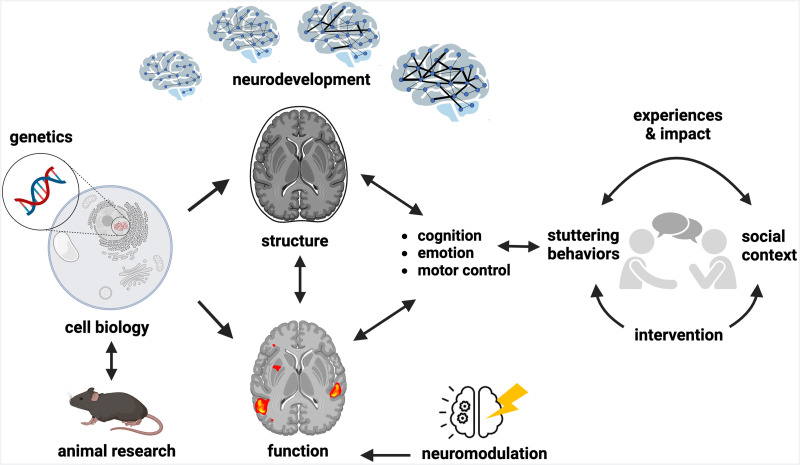
Key areas of research highlighted in the National Institute on Deafness and Other Communication Disorders sponsored workshop on stuttering. Stuttering occurs during communicative speech and is often exacerbated by social, cognitive, and linguistic contexts. Like many complex disorders, the neurobiological bases of stuttering have been difficult to uncover to date. Emerging research findings in the areas of genetics, animal research, and neuroimaging research probing brain function and anatomy in children and adults may pave the way for novel treatment development. Newer intervention approaches involving neuromodulation (i.e., brain stimulation) guided by an updated understanding of neural circuits affected in stuttering may help modulate brain function toward alleviation of core speech symptoms. Shifts in perspectives on the treatment goals for stuttering that include a greater emphasis on confident, authentic communication, and societal acceptance toward stuttering were also topics of interest (more details in the text). Adapted from the University of Delaware Neurobiology of Speech and Language Lab’s web page “Research Strategies” (2025; available at sites.udel.edu/brainspeak/research/), with permission from Dr. Ho Ming Chow.

In June 28–29, 2023, the National Institute on Deafness and Other Communication Disorders (NIDCD) conducted a virtual 2-day workshop titled Stuttering: Our Current Knowledge, Research Opportunities, and Ways to Address Critical Gaps (NIH, 2023). The meeting convened research leaders, representatives from communication disorders departments in academic institutions, patient advocacy groups, clinicians actively working with adults and children who stutter, and funding and regulatory agencies. The participants were selected based on expertise in the field and ability to provide diverse views on current and future priorities for stuttering research. The majority of speakers were selected from among current NIDCD-funded researchers who represent broad and complementary areas of stuttering research. This approach was chosen to capture the profile of work receiving federal funding priority. It was recognized that this constraint would provide a general, but not comprehensive, survey of research progress in stuttering. Two additional international researchers (NEN, KW) who were not being funded by the NIDCD were invited based on their substantial published expertise in novel intervention research projects targeting brain plasticity (see Figure 1 for the key topics presented in this workshop). The final list of speakers was determined by the workshop planning committee led by workshop host Lana Shekim, with co-chairs Nan Bernstein Ratner and Soo-Eun Chang.

The overall goals of the workshop were to (1) outline the current understanding of the biological bases of stuttering, markers of its persistence and recovery across early childhood, review motor, cognitive, and social factors known to relate to stuttering profiles, and discuss treatment approaches in childhood and beyond; (2) identify potential gaps and limitations in current research and treatment research programs; and (3) generate potential future research priorities. Multiple speakers addressed these general themes in conceptually organized sessions. Session organization promoted vigorous discussion around key topic areas, facilitated by short talks given by invited speakers. This article compiles written summaries of the talks as well as proposed priority areas for future research within each topic area provided by the speakers, incorporating perspectives that emerged following group interaction, both following individual breakout sessions and final closing discussion. As this is a summary of a meeting specifically designed to highlight NIDCD-funded research on developmental stuttering, this report is not intended to provide an overview of all areas of research currently conducted in the field of stuttering. However, its themes represent a broad sampling representative of current research orientation to improved understanding of the disorder as it was informed by research up to that point (June 2023).

The second day of the workshop continued with talks focused on intervention and treatment goals, and then shifted to closing breakout sessions with prompts for discussion that were held in small groups. Diverse viewpoints around research priorities and therapeutic approaches in stuttering arose during these final discussions, reflecting cumulative discussion across the two days, and converging ideas reported by multiple groups are summarized at the end of this article.

## KEY RESEARCH AREAS IN STUTTERING

In the sections below, summaries are provided by key topic areas covered during the workshop, followed by priority questions and topics for future investigations.

### Biological Bases of Stuttering

#### Progress in understanding the genetics of stuttering

Over many years, multiple lines of evidence continue to demonstrate that genetics have a large impact on stuttering risk (Ambrose et al., 1993, 1997; Yairi et al., 1996). Estimates of the rate of concordance of stuttering in identical twins have ranged from 38% to 62% (Fagnani et al., 2011; van Beijsterveldt et al., 2010). Heritability (the proportion of variance in a trait that can be attributed to genetics) estimates for stuttering have ranged from 0.42 to 0.82, indicating that both genetic and non-genetic factors influence stuttering (Dworzynski et al., 2007; Fagnani et al., 2011; Ooki, 2005; Rautakoski et al., 2012; van Beijsterveldt et al., 2010).

Many studies have sought to explain the heritability of stuttering by performing family-based genetic analyses and genetic studies of population isolates. Such communities formed due to geographic, cultural, and/or social isolation demonstrate greater genetic homogeneity and thus can help identify the genetic basis of rare conditions that might be more prevalent in these populations. Such studies involving population isolates with relatively higher prevalence of stuttering have identified six candidate causal genes with large effect size within specific groups. A case-control association study in the Han Chinese population identified risk and protective alleles in the *DRD2* gene that encodes for the dopamine receptor D2 protein (Lan et al., 2009). Drayna and colleagues (Kang et al., 2010) identified variants in three genes (*GNPTAB*, *GNPTG*, *NAGPA*) known to disrupt signals that direct enzymes to their target location within the lysosome in consanguineous Pakistani families with familial clustering of stuttering (Drayna & Kang, 2011). Another study identified rare variants within *AP4E1*, a gene involved in intracellular trafficking, in Pakistani and Cameroonian stuttering individuals using a candidate gene sequencing approach (Raza et al., 2015). Additionally, variation within *CYP17A1*, a gene crucial for synthesizing steroid hormones, was associated with stuttering susceptibility in a case-control study of a Kurdish population aged 3–9 years old in western Iran (Mohammadi et al., 2017).

Further studies have attempted to provide insight into how these candidate genes may impact brain structure or function. At least a third of the approximately 20,000 different genes that make up the human genome are active (expressed) primarily in the brain, the highest proportion of genes expressed in any part of the body. *Gene expression* involves the production of functional molecules, such as proteins and non-coding RNAs, which interact with regulatory DNA sequences like enhancers and silencers to control gene activity. While proteins are undoubtedly central to cellular activity, the regulatory network within a cell includes non-coding RNAs, metabolites (such as lipids), and various signaling molecules. Changes in the cellular regulatory pathways combined with environmental factors, can disrupt neural circuits, and contribute to disease risk. In the brain, the components of this regulatory network work together to shape development, plasticity, and function, forming neural circuits that ultimately determine how we move, think, feel, and behave. Research on gene expression in stuttering builds on the idea that altered gene function may have the most significant impact in brain areas where these genes are active. The microarray-based gene expression profile in the human brain collected by Allen Institute for Brain Science (Hawrylycz et al., 2012) has been used to compare the patterns of gene expression variation and stuttering-associated anomalies across the brain. For instance, Chow et al. (2020) found that expression patterns of *GNTPG* and *NAGPA* in the brain showed a significant spatial correlation with brain regions where group differences were found in gray matter volume, suggesting a potential mechanism by which the genes in stuttering may lead to its symptoms (Chow et al., 2020). Similarly, Benito-Aragón et al. (2020) found that expression of *GNTPG* was spatially correlated with cortical networks that have been shown to be involved in stuttering (Benito-Aragón et al., 2020). Furthermore, Chow et al. (2021) found that, compared to controls, participants who stutter with rare heterozygous *AP4E1* variants showed reduced grey matter volume in the thalamus, visual areas, and the posterior cingulate cortex as well as reduced fractional anisotropy in the corpus callosum, brain areas where gene expression of *AP4E1* is relatively high (Chow et al., 2021). Together, these studies suggest the importance of these lysosomal storage genes in brain regions that have been implicated in stuttering. However, the intricate relationship between gene regulation, brain circuits, and behavior poses significant challenges, underscoring the need for *direct* mechanistic studies to reveal how these genes may influence neuroimaging phenotypes and ultimately increase the risk of stuttering.

Despite these studies of potential gene function in isolate communities, subsequent candidate gene replication studies have failed to replicate findings in other families or general populations, suggesting that additional genetic effects on stuttering risk have yet to be identified. Most genetic studies of stuttering have failed to reveal broadly relevant monogenic causes, supporting the hypothesis that stuttering in most populations is polygenic, with multiple contributing genetic factors. Thus, characterization of the specific genetic architecture of stuttering in general populations is still emerging.

In particular, genome-wide association studies, or GWASs, test the effect of common variants across the genome on a trait or condition in a sample from a general population. Compared to family studies, GWASs require much larger sample sizes (often in the hundreds of thousands or millions) to reach significance while accounting for multiple test correction and discovery of more modest effects. A central challenge to performing GWASs of stuttering is amassing sufficiently sized groups that include both genetic (genotype) and trait (phenotype) data for well-powered analyses. The recent development of large-scale genetic databases linked to participants’ electronic health records or survey data offer a cost-effective method for exploring genotype–phenotype relations at scale.

However, stuttering is underrepresented within electronic health records compared to generally reported population prevalence (Pruett et al., 2021). To address the sample size challenge, the first published GWAS of stuttering was performed on a proxy stuttering phenotype derived using machine learning of electronic health record data linked to a DNA databank (Shaw et al., 2021). The model took a series of diagnostic billing codes that occur within the medical records of people who stutter more often than expected by chance and identified additional people with this stuttering-associated phenome in a genotyped patient population (Pedregosa et al., 2011). A GWAS of this stuttering-related measure (9,239 predicted cases and 83,503 controls) identified a genome-wide significant association close to the gene *FAM49A* (also known as *CYRIA*; CYFIP related Rac1 interactor A) in participants of predominantly European ancestry. *FAM49A* is highly expressed in stuttering-relevant tissues such as the central nervous system (cerebral cortex, basal ganglia) as well as the thyroid gland, granulocytes, and monocytes (Uhlén et al., 2015). In addition, there was a near-significant association within *ZMAT4* (zinc finger matrin-type protein 4) in participants of African ancestry. *ZMAT4* is also highly expressed in the central nervous system, especially in tissue types present in the cerebral cortex, cerebellum, and hippocampus, and is modestly expressed in the basal ganglia (Uhlén et al., 2015).

An additional GWAS of stuttering was performed using data from the clinically ascertained, multiethnic, International Stuttering Project and the National Longitudinal Study of Adolescent to Adult Health (Add Health), which was survey-based. The study meta-analyzed genotype data from the International Stuttering Project (1,345 clinically ascertained cases and 6,759 controls) and Add Health (785 self- or parental reported cases and 7,572 controls; Harris et al., 2019; Polikowsky et al., 2022). One genome-wide significant association was observed within *SSUH2*, which to date has a reported role in odontogenesis (tooth development; Polikowsky et al., 2022).

The largest GWAS of stuttering to date was performed using a genetic database of participants from 23andMe. These ancestry- and sex-stratified analyses of self-reported stuttering comprised more than 1 million individuals in total (99,776 cases and 1,023,243 controls) and identified 57 unique associations (87 total across all sex- and ancestry-stratified analyses) after multiple test correction (Below et al., 2023). Many of the identified associations for stuttering risk were near genes previously associated with other neurological traits, strengthening the assumption of a neurological basis for stuttering. For example, *VRK2* has been associated with depression (Turley et al., 2018), neuroticism (Luciano et al., 2018), and schizophrenia (Li et al., 2017). *SEMA6D* is involved in pathways related to nervous system development and ADHD (Martin et al., 2018), and *SLC39A8* is a gene associated with brain volume measurements (Lam et al., 2019; Zhao et al., 2019), in addition to a wide range of other traits. Follow-up genetic analyses were also performed to validate whether the genetic architecture captured by the self-reported stuttering data reflects the genetic architecture of clinically diagnosed stuttering. Specifically, polygenic risk scores, or a weighted sum of the number of risk variants, were calculated from the self-reported 23andMe dataset and significantly predicted stuttering in the International Stuttering Project, a clinical cohort. Overall, results from these three GWASs provide additional evidence that stuttering is a polygenic trait, potentially influenced by both rare and common genetic variation. While GWASs help identify common genetic variants across populations, family studies focus on sharing of rare, high-impact mutations within affected families. The intersection of these two approaches offers a powerful avenue for validating genetic findings across different cohorts. By identifying shared genetic pathways, GWAS can complement family-based findings and focus research efforts on key variants associated with stuttering.

Together, these studies provide insights into genetic factors underlying stuttering and represent steps forward in our understanding of the condition. Similar to other neurological conditions such as Alzheimer’s or Parkinson’s disease, identified genetic variants may not fully explain the disorder in the general population, which can introduce challenges for translational research. Translating genetic factors into interpretable biological processes that directly impact trait development or risk remains highly complex. Thus, the integration of functional investigations using multifaceted interrogation from computational, cellular, and animal model approaches are needed.

#### Animal models

The challenge of understanding how diverse genetic risk factors contribute to stuttering’s varied symptoms and severities lies in the need for detailed studies at the molecular, cellular, and circuit levels, which cannot currently be conducted noninvasively in humans. Animal models have proven to be invaluable tools in advancing our understanding of the neurogenetic mechanisms at the cellular and circuit levels in health and disease. In fact, recent findings from brain connectome reconstructions across more than 120 mammalian species have indicated a fundamental principle governing brain network connectivity: ensuring consistent global communication despite variations in the network’s specific architecture (Assaf et al., 2020). This insight supports the potential use of animal model research to uncover fundamental circuit mechanisms, including those governing vocal behaviors.

Although some data from avian species indicate their suitability as models of stuttering (Chakraborty et al., 2017; Kubikova et al., 2014; Moorman et al., 2021), one of the most significant recent advances in stuttering research is the generation of mouse models with variants in the *Gnptab* gene that are linked to stuttering in humans (Barnes et al., 2016). To create these models, Drayna and colleagues (see Barnes et al., 2016) engineered mice to carry mutations such as a specific missense mutation where the amino acid glutamic acid (Glu) at position 1179 is substituted with lysine (Lys) in the *Gnptab* gene (Glu1179Lys). This mutation corresponds to the Glu1200Lys mutation found in humans who stutter (Barnes et al., 2016). These genetically modified mice are then studied for alterations in their ultrasonic vocalizations, which serve as a proxy for human speech. The mutant mice exhibit distinct vocalization patterns, such as fewer vocalizations and longer pauses between vocalizations (Barnes et al., 2016). Recent data using this mouse model has already led to a new hypothesis suggesting that astrocytes, which are star-shaped nonneuronal brain cells (glia cells), may play a key role in the development of stuttering disorders (Adeck et al., 2024; Han et al., 2019; Maguire et al., 2021; Turk et al., 2021). Strikingly, the morphometric analysis suggested that the astrocytes within the vocal motor circuits are smaller and have reduced complexity in the mouse model of stuttering (Adeck et al., 2024). Similar morphometric deficits were also seen in microglia, the primary immune cells residing within the parenchyma of the nervous system, but these differences were mainly observed in brainstem regions, where motor nuclei that innervate speech muscles are located (Adeck et al., 2024). These differences in cellular architecture of glia cells likely affect their functions. Future work is needed to elucidate the functional significance of the *Gnptab* variant in glia cells.

So far, there are only two mammalian models for developmental stuttering: the fully validated *Gnptab*-mutant mouse and a recently developed mouse model with a mutation in the CYP40, a chaperone protein (Morgan et al., 2023). In other neurodevelopmental disorders, such as autism spectrum disorder (ASD) and attention deficit hyperactivity disorder (ADHD), multiple animal models have been developed to study each disorder. For instance, 16 and 14 rodent models (including eight transgenic mouse models) have been used to study the possible mechanism(s) underlying ASD and ADHD, respectively (Ergaz et al., 2016; Rahi & Kumar, 2021; Regan et al., 2021; Sagvolden, 2000). Using imaging experiments on the existing 16 mouse models for ASD distinguished four distinct subtypes for this disorder (Zerbi et al., 2021), which were later validated in human studies (Buch et al., 2023). Although animal models have their limitations, availability of multiple animal models will enable stuttering research to study different aspects of brain development, define possible subtypes for stuttering, and distinguish contributions of the discovered genes to the pathophysiology at the cellular and circuit levels (SheikhBahaei et al., 2023).

#### Interim discussion: Research priorities relevant to biological bases of stuttering

- Genetic analyses that disentangle sex-specific demographic features of stuttering across the lifespan and recovery- and persistence-specific effects are needed.
- Development of innovative animal models that utilize recently identified genes associated with stuttering are needed to provide deeper insights into the genetic underpinnings and mechanisms of stuttering.
- Large-scale genetic analyses of stuttering with robust and detailed clinical phenotyping are needed to identify effects associated with severity and as well as potential sub-phenotypes, especially in populations that are minoritized and/or historically underrepresented in genetic research.
- Population-based and additional family-based analyses of rare-variant effects in stuttering are needed and are likely to identify additional genes impacting risk, especially in populations with reported high stuttering prevalence, such as Australians (Reilly et al., 2009, 2013).
- Transcription-wide association studies can facilitate downstream analyses for interpretation of identified genetic effects. In addition, annotating variant effects on their roles in regulating phenotypes that can be measured with neuroimaging will likely be hypothesis-generating for downstream functional studies in both animal models and human studies.
- Animal models can be used to dissect the complex genetic factors that may influence the sex-specific characteristics of stuttering.
- Animal model studies can be incorporated into the larger body of basic and clinical research in stuttering to enhance our understanding of biological underpinnings that may lead to improved treatment approaches, including pharmacological interventions.

### Demographic, Physiological, and Neural Bases of Stuttering Persistence and Recovery in Childhood

#### Demographic and physiological factors

Approximately 5%–11% of children go through a period of stuttering in the preschool years; however, unassisted recovery is frequent (Bloodstein et al., 2021). Given this relatively high recovery rate, a significant concern is how to differentiate children whose stuttering is more likely to resolve from children whose stuttering is more likely to persist. As discussed later, this is extremely relevant to powering and interpreting studies of early intervention, among other practical as well as basic science questions. At the individual level, children who continue to stutter are at risk for developing negative attitudes toward communication and psychological distress that may adversely affect their lives. Thus, discovering risk factors to predict stuttering persistence affords insight into the underpinnings of chronic stuttering and allows us to intervene earlier to prevent the development of adverse impact.

To understand the underpinnings of complex disorders like stuttering, researchers have adopted multifactorial research approaches to examine how the onset and course of stuttering co-occurs with dynamic changes in other aspects of development during early childhood. These approaches have revealed key demographic, physiological, and neurophysiological factors associated with greater risk for stuttering persistence. Children with a positive family history of stuttering into adulthood have a higher probability of persisting (Singer et al., 2020; Walsh et al., 2021; Yairi & Ambrose, 2005) and males are more likely to persist than females (Singer et al., 2020; Walsh et al., 2018). Most children recover from stuttering within approximately 18 months of onset, so it follows that the longer a child has been stuttering, the less likely they are to recover (Singer et al., 2022; Yairi & Ambrose, 2005). Finally, children who are older than approximately 4 years of age when they begin to stutter may be at higher risk for persistent stuttering (Singer et al., 2020).

Kinematic measures of speech motor performance have shown less consistent articulatory patterning during speaking tasks in children who stutter compared to children who do not stutter (MacPherson & Smith, 2013; Usler et al., 2017; Walsh et al., 2015), and emerging data suggest differences in rhythm discrimination and production in children who stutter (Erdemir et al., 2023; Wieland et al., 2015). Clinical indices, such as children’s speech sound production abilities (Singer et al., 2020; Walsh et al., 2021) and nonword repetition performance, which accesses phonological working memory as well as other processes, are sensitive predictors of stuttering recovery and persistence (Spencer & Weber-Fox, 2014; Walsh et al., 2021). Finally, preschool children who experience more frequent stuttering events may be at higher risk for persisting (Walsh et al., 2020). As a next step, researchers have explored whether combinations of clinical and demographic risk factors confer an increased risk of stuttering persistence (Singer et al., 2022; Walsh et al., 2021). This work is a stepping stone toward identifying individual children at greater risk for stuttering persistence in order to better prioritize therapy resources, inform the selection of treatment targets, and disentangle assisted from spontaneous recovery in the evaluation of treatment effectiveness.

#### Neuroanatomical and neurophysiological factors

A recent study showed that developmental trajectories in brain morphology seem to differentiate children who recover from those who go on to have persistent stuttering. In this longitudinal study (Chow, Garnett, Koenraads, & Chang, 2023), children with persistent stuttering and those who recovered were compared with age-matched peers who do not stutter to examine the developmental trajectories of both gray and white matter volume during the preschool and school age years. A total of 470 magnetic resonance imaging (MRI) scans were analyzed from 95 children who stutter (72 persistent and 23 recovered) and 95 peers who do not stutter between 3 and 12 years of age. Each participant was scanned up to four times, with an average inter-scan interval of 1 year. Overall group differences in gray and white matter volume and their growth rate differences with age were compared in preschool age (3–5 years old) and school age (6–12 years old) children. Potential effects of sex, IQ, intracranial volume, and socioeconomic status were controlled for in the analyses. Children who would eventually go on to persist in stuttering exhibited significant decreases in gray matter volume in cortical and subcortical areas of the basal ganglia thalamocortical circuit, including in the putamen and left premotor cortex. Children who would go on to recover from stuttering showed normalization or compensation of earlier occurring structural changes affecting basal ganglia thalamocortical circuit structures. Children who would go on to recover from stuttering also showed white matter volume increases in regions coinciding with major white matter tracts that interconnect key gray matter areas in this neural circuit. Results corroborated earlier published reports also suggesting that increased major white matter tract development and possible strengthening of structural connectivity encompassing auditory-motor and cortico-basal ganglia networks might be associated with natural recovery from stuttering. These findings point to possible core deficits in the basal ganglia thalamocortical circuit that may underlie stuttering onset and persistence, and a normalization of these circuits that may be associated with recovery.

Stuttering is more likely to occur and is most apparent during spontaneous speech production in a social communicative context. However, due to the negative effects of speech-related movement artifacts on many neuroimaging techniques, most previous brain imaging studies of stuttering have employed relatively simple speaking tasks (e.g., single-word production) or nonspeaking contexts (e.g., wakeful rest) to examine brain functions. Functional near-infrared spectroscopy (fNIRS) is less sensitive to movement artifact and, as such, has been used to examine cortical activation during overt speaking tasks. Hosseini et al. (2018) used a sparse learning framework to discover whether cerebral hemodynamic features from fNIRS recording of speech production could differentiate groups of children who do not stutter, children with persistent stuttering, and children who recovered from stuttering. They found that children who recovered from stuttering had patterns of brain activity during speech production similar to those of their peers who do not stutter, while children with persistent stuttering showed atypical brain activity in channels spanning the left inferior frontal gyrus. These neurophysiological biomarkers accurately distinguished children who persisted from the other two groups of children (Hosseini et al., 2018).

Chow and colleagues (Chow, Garnett, Bernstein Ratner, & Chang, 2023) developed a novel method to successfully circumvent speech-related artifacts that occur during functional MRI (fMRI) recording. This fMRI denoising technique permits the study of children’s neural processes associated with continuous speech production and can identify the neural patterns underlying persistent stuttering. Results showed that, relative to children who do not stutter, children who stutter exhibited reduced activation of the left dorsal premotor area, which was particularly evident during spontaneous speech production compared to automatic (nonpropositional, overlearned) speech production. This reduction was greater with increased age in children who stutter. Age-related reductions were also observed in bilateral dorsal premotor cortex, left thalamus, and left putamen when children who stutter *prepared* to initiate continuous speech. These results further support the proposal that stuttering is associated with impairments in the basal ganglia thalamocortical circuit.

Finally, in the absence of overt speech production, studies have confirmed delayed or atypical event-related brain potential (ERP) responses during receptive language tasks indexing semantic, syntactic, and phonological processing in children whose stuttering persists (Hampton Wray & Spray, 2020; Kreidler et al., 2017; Mohan & Weber, 2015; Usler & Weber-Fox, 2015). These studies point to a lag in the neurodevelopment of cortical substrates of language for a proportion of children who persist in stuttering.

#### Interim discussion: Research priorities relevant to childhood persistence and recovery

- Longitudinal studies can help identify how dynamic interactions among demographic, physiological, and clinical variables associated with stuttering may influence persistence or recovery in an individual child who stutters.
- Frameworks that have been used to successfully identify risk factors associated with stuttering persistence can be further applied to address the development of the adverse impact of stuttering in children who persist.
- More research is needed that examines neural responses associated with stuttering in ecologically valid contexts, such as during spontaneous speech and conversation in both children and adults.
- Relationships between structural and functional brain patterns in people who stutter, and differences between children whose stuttering persists and those who recover from stuttering, need to be better delineated.
- Discovery of neurological/neurophysiological biomarkers is expected to help distinguish among subgroups of people who stutter, including children who persist or recover.
- Further work is needed to examine molecular and cellular mechanisms associated with the development of structural and functional brain differences in people who stutter.
- Sex differences in brain developmental trajectories that are linked to persistence and recovery of stuttering in childhood and adolescence are still understudied.

### Neural Bases of Stuttering in Adults

#### Variations in brain structure and function among adults who continue to experience stuttering

**Figure 2.**
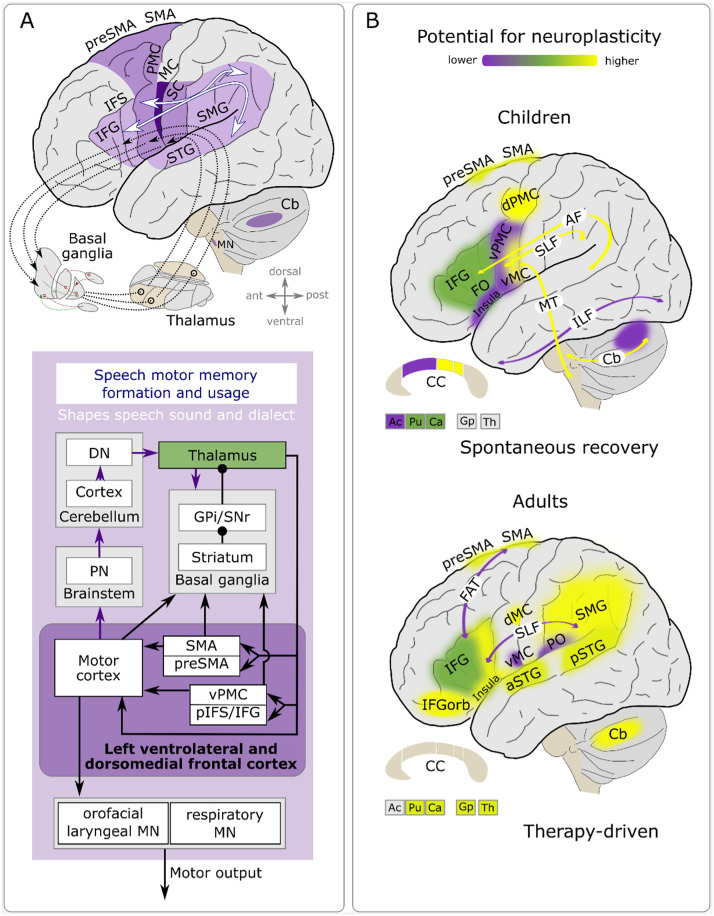
Simplified qualitative summary of the imaging literature. Reorganization potential depends on multiple factors, and evidence levels vary across individual brain regions. Brain correlates for speech motor memory formation and usage (A) and brain correlates for structural reorganization associated with spontaneous recovery in children and for functional reorganization associated with therapy-induced improvement of speech fluency in adults who stutter (B). *Abbreviations*: Ac = nucleus accumbens; AF = arcuate fasciculus; aSTG = anterior superior temporal gyrus; Ca = caudate nucleus; Cb = cerebellum; CC = corpus callosum; dMC = dorsal primary motor cortex; DN = dentate nucleus; dPMC = dorsal premotor cortex; FAT = frontal aslant tract; FO = frontal operculum; Gp = globus pallidus; Gpi = globus pallidus internal segment; IFG = inferior frontal gyrus; IFGorb = inferior frontal gyrus pars orbitalis; IFS = inferior frontal sulcus; ILF = inferior longitudinal fasciculus; MC = primary motor cortex; MN = vocal tract motor neurons; pIFS = posterior inferior frontal sulcus; PMC = premotor cortex; PN = pontine nuclei; PO = parietal operculum; preSMA = presupplementary motor area; pSTG = posterior superior temporal gyrus; Pu = putamen; SC = primary somatosensory cortex; SLF = superior longitudinal fasciculus; SMA = supplementary motor area; SMG = supramarginal gyrus; SNr = substantia nigra pars reticulata; STG = superior temporal gyrus; Th = Thalamus; vMC = ventral primary motor cortex; vPMC = ventral premotor cortex. Adapted from Neef and Chang (2024).

Due to methodological challenges, neuroimaging findings in children who stutter (Chang & Zhu, 2013; Chang et al., 2008, 2015, 2016; Koenraads et al., 2019) are still very recent, and deeper insights are expected from ongoing and future studies. To date, imaging technology has been more extensively exploited in the examination of adults who stutter. Numerous neuroimaging studies with adults who stutter have associated alterations in basal ganglia, cerebellar, and cortical sensorimotor and fronto-parieto-temporal regions with persistent stuttering, as synthesized in several reviews (Chang et al., 2019; Neef et al., 2015; Watkins et al., 2016). Involved brain structures are characterized by irregular activity, connectivity, or morphology and reflect neuroanatomical hubs of large-scale brain networks involved in speech production (Figure 2A). More specifically, imaging findings link persistent stuttering with brain regions involved in speech motor memory formation, speech initiation, sequencing and timing, sensorimotor integration, and speech motor execution (Neef & Chang, 2024). Thus, brain imaging reveals a widespread array of brain activity differences, underscoring the complexity of the neural underpinnings of stuttering. Stuttering is characterized by alterations in several speech-related interconnected brain networks, highlighting its nature as a multifaceted neurological disorder rather than a condition confined to a single brain region. However, brain differences go beyond the speech production network. They occur in a broader range of neural circuits, including those involved in cognitive, language, and emotional processing. This comprehensive profile of neural divergence suggests that stuttering is a complex condition influenced by a wider array of brain functions than previously thought. One example is increased activity (Belyk et al., 2015; Brown et al., 2005; Neef et al., 2015) and connectivity (Neef et al., 2018) of the frontal homolog of Broca’s region in the right hemisphere, including pars opercularis, pars triangularis, and pars orbitalis. Initially, such hyperactivity was discussed in light of compensatory mechanisms (Kell et al., 2009; Preibisch et al., 2003). Advances in the cytoarchitectonic and neurofunctional partitioning of the cortex form the basis for a discussion of this hyperactivity in terms of functions specifically performed by the right frontal cortical subregions of the inferior frontal gyrus, including action control, action inhibition, conflict resolution, cognitive reasoning, and social-emotional processing (Hartwigsen et al., 2019). In this context it is important to note that adults who stutter can exhibit a highly variable phenotype, with a rich repertoire of overt stuttering symptoms, as well as covert strategies to avoid and evade stuttering. With this in mind, it is clear that the findings from brain imaging studies of structure and function in adults who experience lifelong stuttering make it more difficult to decipher the causes and consequences of the condition, when contrasted with the study of children who stutter. Future neuroimaging studies might benefit from identifying and differentiating subtypes of stuttering and people who stutter.

In addition to the aforementioned studies exploring differences in the macrostructure of brain areas in people who stutter, along with how these areas vary in terms of their functional and structural connections, microstructural quantitative imaging techniques are beginning to provide new information. For example, analysis of quantitative MRI brain scans obtained in a large sample of adult men and women who stutter revealed that the putamen and left speech motor cortex had higher iron content (Cler et al., 2021) than that seen in adults who do not stutter. This higher level of iron in gray matter could be an indication that dopamine levels are higher in stuttering, which is consistent with some neurobiological theories (Alm, 2021). Higher iron is also found in association with lysosomal dysfunction, and as noted earlier, causal genetic variants identified in stuttering are involved in the lysosomal pathway (Frigerio-Domingues & Drayna, 2017). Furthermore, the brain regions where higher concentrations of iron were found in adults who stutter are spatially coincident with the brain regions where these genes are highly expressed (Benito-Aragón et al., 2020). It is worth noting that iron concentration increases with age and did so in both people who stutter and people who do not stutter (Cler et al., 2021). However, group differences were evident even at the youngest ages in the study, namely the late teens. This finding needs to be replicated and studies of younger age groups as well as longitudinal investigations are needed.

#### Interim discussion: Research priorities relevant to persistent stuttering in adulthood

- Further work is needed to determine whether different brain differences repeatedly observed in cross-sectional studies of adults who stutter are likely to reflect either the causes or consequences of stuttering.
- Findings that suggest increased iron concentration in the brains of people who stutter will need to be replicated in larger samples and at earlier ages followed longitudinally.
- Animal models of stuttering will be valuable to test whether removal of iron affects vocal production quality and behavioral analogs of stuttering in humans.
- Large study samples may potentially allow for the identification of neural fingerprints in subgroups of adults who stutter, facilitating personalized treatments, such as neuromodulation.

#### Impact of stuttering interventions on adult brain function

Neuroimaging provides a window into whether therapeutic interventions may engage brain circuits of the speech motor system in order to enhance speech fluency. Determining potential brain structures involved in the neural architecture of stuttering can enhance the neurobiological understanding of speech motor circuits and mechanisms that are involved in the disorder. Over many years, six independent labs set out to test brain changes associated with a wide array of stuttering therapies and revealed functional patterns of reorganization within and beyond the speech network. Tested interventions involved speech restructuring therapy (De Nil et al., 2001, 2003; Kell et al., 2009, 2018; Korzeczek et al., 2021; Neef et al., 2022; Neumann et al., 2005, 2018; Preibisch et al., 2003), fluency-enhancing techniques, such as metronome paced speaking (Toyomura et al., 2015), metronome-timed speech/choral speech with transcranial direct current stimulation (tDCS; Chesters et al., 2018, 2021), and pharmacological treatment with Risperidone, a D_2_ receptor/5HT_2_ receptor antagonist (Maguire et al., 2021). Although the interventions had no statistically significant impact on brain morphology, they did lead to observable changes in brain activity. Activity changes were observed in brain regions and circuits that support speech-related auditory-motor processes and speech motor learning in areas such as the cerebellum, cortico-basal ganglia circuits, and cortico-cortical circuits, including the dorsal motor cortex, inferior frontal gyrus, insula, supplementary motor area, supramarginal gyrus, and posterior superior temporal gyrus (Figure 2B). However, it should be noted that these findings come from a small number of studies with limited sample sizes and statistical robustness, and different methods of analysis. Consequently, their comparability is somewhat limited. For example, in task-related fMRI designs, speech tasks frequently lack specificity, and findings are often limited to brain activity during word or sentence reading, rather than conversational speech. This limitation complicates the process of reaching conclusive findings about essential mechanisms related to typical goals of stuttering interventions, such as speech motor learning, rhythm and prosody production, and automatization. Optimization of fMRI paradigms is needed, as well as an expansion of the spectrum of methods to include electroencephalography and magnetoencephalography, in order to expand knowledge of the neuroplasticity potential of stuttering therapies and to support or establish the rationale for advanced therapeutic tools, such as brain stimulation or pharmacological treatments for adults who stutter. Moreover, there is a need for additional research to explore therapy-induced brain changes in children and adolescents who stutter, as this population holds a greater potential for neuroplasticity. Additionally, it remains unclear which specific time periods are crucial for achieving sustainable modulation of the neural correlates linked to fluent speech production.

#### Interim discussion: Research priorities relevant to interventions that affect brain function

- There is a need to develop sophisticated intervention paradigms and approaches that address specific neural circuits and mechanisms.
- Critical periods for the assisted neural reorganization of the speech motor system in children and adolescents who stutter need to be identified.

#### New interventions that seek to alter brain connectivity to enhance fluent speech

Efficacious interventions for adults who stutter who choose to pursue therapy to change their fluency profiles are few, require considerable effort, and show limited long-lasting change. Noninvasive brain stimulation could potentially provide an effective and useful adjunctive tool to enhance speech fluency training in adults who stutter (Busan et al., 2021). Two forms of stimulation have been evaluated in people who stutter: transcranial magnetic stimulation (TMS) and tDCS. TMS involves bulky, expensive, and specialist equipment to excite or inhibit cortical areas. Two reports of single cases and one small sample of eight adult men who stutter describe mixed results in terms of the efficacy of this form of stimulation in enhancing fluency (Bakhtiar et al., 2024; Le Guilloux & Compper, 2018; Mejías & Prieto, 2019; Tezel-Bayraktaroglu et al., 2020). Transcranial current stimulation (including direct or alternating current), if shown to be effective, appears to offer more promise as a tool to augment speech training interventions in the clinic. This method uses relatively inexpensive and safe equipment and requires minimal training. Of benefit to treatment outcome study designs, it is also possible to have an effective sham or placebo condition in transcranial current stimulation studies and to conceal the nature of the stimulation (i.e., whether it is real or sham) from both the participant and the researcher. The efficacy of this method was demonstrated in a double-blind randomized controlled trial using tDCS paired with speech fluency training in people who stutter (Chesters et al., 2018). Two small groups of 15 men with at least a moderate level of stuttering (according to the Stuttering Severity Instrument–4; Riley, 2009) received five 20-minute daily sessions of either 1-mA anodal or sham stimulation over the left inferior frontal gyrus while fluent speaking was temporarily induced using metronome-timed speech or choral reading. When tested 1 week after the end of the 5-day treatment, the group that received anodal stimulation showed significant reductions in the frequency of disfluencies and this effect persisted 6 weeks later during reading, but not in conversational speech samples. The group that received sham stimulation, an effective placebo, showed no changes in their levels of disfluency. Brain imaging data acquired before and 1 week after the training showed increases in speech-evoked activity during fluent speech production in the dorsal striatum and medial and left inferior frontal cortex, but only in the group who had the anodal stimulation, not in the group who received sham treatments (Chesters et al., 2021). Similar behavioral effects were recently observed in a larger sample of people who stutter who received anodal tDCS over left superior temporal cortex while fluency was induced with delayed auditory feedback (Moein et al., 2022). Together, these findings suggest that noninvasive brain stimulation may be a potential adjunct to support fluency training efforts in adults who stutter who seek treatment. Another study demonstrated positive effects of cathodal (inhibitory) stimulation over right inferior frontal cortex in reducing disfluencies for reading during a single stimulation session in a small group of 13 adults who stutter (Yada et al., 2019).

It is worth noting, however, that further investigation is needed. Not all studies demonstrate efficacy of different brain stimulation protocols in enhancing fluency in adults who stutter. For example, in 14 adults (3 women) who stutter, a single session of either active or sham high-definition direct current stimulation targeting the left supplementary motor area was shown to reduce stuttering-like disfluencies during reading but not conversation based on measurements collected before and after stimulation (Garnett et al., 2019). There were no differences in the effects of active and sham stimulation on neural activity during continuous speech (reading), but active stimulation did significantly modulate the relationship between stuttering severity and brain activity in the right thalamocortical network (Garnett et al., 2019).

It is also worth highlighting the difficulties inherent in these studies in terms of studying large samples of people who stutter over several weeks and months with multiple sessions for both intervention and assessment of outcomes. There is significant within-subject and between-subject variability in stuttering symptoms. It may be necessary to restrict investigations to those with high stuttering severity to maximize the power to detect change. There is also a high likelihood of publication bias (only positive findings reported) or the file-drawer problem (negative findings not reported). Considerable work is required both to replicate this fledgling work and to further understand the neural mechanism(s) by which noninvasive brain stimulation can support fluency training. Once efficacy of stimulation to enhance fluency is established, optimization of stimulation parameters is required, including specifying stimulation sites, electrode montages, dose, and frequency of training sessions.

#### Interim discussion: Research priorities relevant to neuromodulation interventions for stuttering

- Replication studies are needed to establish effective fluency enhancement by noninvasive brain stimulation in larger samples.
- Extended research is required to permit evaluation of effects in different subgroups or subtypes of stuttering.
- Research should aim to optimize and systematize protocols for noninvasive brain stimulation to determine target brain areas for stimulation as well as dose and frequency of sessions that lead to clinically meaningful changes for adults who stutter who seek therapies to change their fluency profiles.
- Further work is required to better understand the mechanisms of action of brain stimulation protocols shown to alter spoken fluency in adults who stutter.

### Understanding Speech Motor Control in Stuttering

#### Auditory–motor integration

One challenge for research on the sensorimotor basis of stuttering is identifying which of the many motor, sensory, and neural differences between stuttering and nonstuttering speakers may be causally related to the overt symptoms of the disorder. Behavioral and neuroimaging evidence indicates that processes related to *auditory prediction* and *auditory–motor integration* form one of the most promising lines of inquiry. Although the somatosensory system is also essential for fast and accurate speech motor control, there is—at the present time—not nearly as much evidence indicating that it plays a key role in the fluency disruptions characteristic of stuttering.

Recent findings regarding auditory–motor integration in children and adults who stutter have informed biologically plausible hypotheses (Max et al., 2004) that are now supported by empirical data on fluent and stuttered speech (e.g., Garnett et al., 2022; Kim et al., 2020), models of the role of subcortical structures, such as the basal ganglia (e.g., Chang & Guenther, 2020), and developing insights into the role of neurotransmitters, such as dopamine (Maguire et al., 2021). However, existing research programs have studied adults more intensively than children, and thus there is a paucity of data concerning the development of auditory–motor integration in children who stutter.

One line of work has focused specifically on sensory predictions related to the movement-related modulation of afferent signals in various sensory modalities, including the auditory system while speaking. These studies demonstrated that typical speakers already show a modulation of auditory processing during movement planning immediately prior to speech onset (Daliri & Max, 2016). However, this pre-speech auditory modulation is minimal or absent in adults who stutter (Daliri & Max, 2015a, 2015b). Interestingly, the difference in the pre-speech auditory modulation between stuttering and nonstuttering adults was no longer statistically significant when speaking with delayed auditory feedback, a condition known to be fluency-enhancing for many individuals who stutter (Daliri & Max, 2018). These demonstrations suggesting that individuals who stutter show atypical pre-speech auditory modulation are highly consistent with other work demonstrating that stuttering speakers show strongly reduced adjustments in speech movement planning based on auditory feedback from previous trials (Daliri et al., 2018; Kim et al., 2020; Kim & Max, 2021). Overall, the explanatory power of these lines of work lies in the fact that the findings directly relate to sensorimotor processes whose disruption or suboptimal functioning could realistically result in the types of speech dysfluencies observed in stuttering (Max & Daliri, 2019).

#### Interim discussion: Research priorities relevant to sensorimotor integration in stuttering

- We need research to examine how differences in auditory–motor integration are related not only to *trait* characteristics of stuttering (i.e., what differentiates stuttering and nonstuttering speakers?) but also, or especially, to its *state* characteristics (i.e., what is their role in stuttering moments?).
- Additional research is needed to explore the exact role of neurotransmitters that are hypothesized to play a role in stuttering (e.g., dopamine) and delineate how those processes change during the alleviation of stuttering in fluency-enhancing conditions.
- More work needs to be conducted involving sensorimotor integration in children who stutter, particularly its role in persistence and recovery.

#### Computational modeling of the speech motor control system

The speech motor control system is thought to consist of two largely distinct circuits for generating speech movements: an *articulation circuit* responsible for sending highly coordinated, time-varying motor commands to the speech articulators (otherwise known as *motor programs*), and an *initiation circuit* that is responsible for turning the motor programs on and off at the right instants in time in order to maintain fluency of speech. Given this conceptualization, computational modeling has the potential to simulate consequences of breakdown in any of these circuits. According to Guenther (2016), stuttering is the result of impaired processing in the initiation circuit, which consists of cortico-basal ganglia-thalamo-cortical loops that monitor sensorimotor context (via cortico-striatal projections) in order to determine the proper instant in time for releasing the next motor program in a sequence of speech gestures (Chang & Guenther, 2020; see also Alm, 2004). Computer simulations with the DIVA (directions into velocities of articulators) and GODIVA (gradient order DIVA) neurocomputational models have been used to explore this concept (Civier et al., 2010, 2013), along with other possible accounts of stuttering. These simulations suggest the importance of future research looking into different subtypes of stuttering that may reflect impairments in different portions of the cortico-basal ganglia loops.

Thus, an area of high potential importance for understanding the neural bases of stuttering is the creation of models that elaborate on function within the basal ganglia circuit and how this function breaks down to result in stuttering. Although alternative proposals exist (e.g., Hickok et al., 2011), the GODIVA model (Guenther, 2016) has been refined explicitly over the past decade to provide an account of how impaired computation in specific structures within the basal ganglia loop can lead to stuttering behaviors. At present, however, this model remains speculative, and ongoing studies involving both behavioral and neural measures of normal and stuttered speech are being carried out to test and refine the model’s account. Of particular interest is electrophysiological data collected during neurosurgery from basal ganglia structures (including the subthalamic nucleus, or STN, and globus pallidus, or GP) and cortical areas involved in speech production; these data provide a rare glimpse into single neuron properties in the human basal ganglia during speech and other tasks. Such data are available because the basal ganglia, in particular STN and GP, are frequently targeted for implantation of deep brain stimulation (DBS) electrodes for the treatment of Parkinson’s disease and dystonia. Although DBS is not currently used to treat stuttering, the fact that the technology can be used to alleviate abnormal processing in the basal ganglia circuit, along with the fact that the technology is already widely and safely used for Parkinson’s fisease and dystonia, suggest that such an approach may be a realistic future option for those with persistent severe stuttering.

#### Interim discussion: Research priorities relevant to computational modeling in stuttering

- Synergistic experimentation and modeling, with experiments aimed at testing key model-based hypotheses may lead to an improved understanding of the mechanisms involved in speech sequencing and their breakdown in stuttering.
- Data specific to distinct stuttering event subtypes (blocks, repetitions, prolongations) and their neural correlates may have value when used to refine neural models of stuttering. Detangling neural correlates of distinct types of stuttering events may be an important step for better understanding the disorder as well as for designing individualized treatments.
- It will be useful to exploit cutting-edge human neurophysiological recording capabilities (such as single unit recording from basal ganglia during DBS surgery) to obtain data for validating computational models of basal ganglia function and its involvement in stuttering.
- We should investigate the potential for DBS as a possible future treatment for severe cases of stuttering.

### Cognitive and Social Aspects of Stuttering

#### Executive function and attention in developmental stuttering

Theories of stuttering have long acknowledged the potential role of cognitive factors, such as executive function and attention, in developmental stuttering (e.g., Postma & Kolk, 1993; Smith & Weber, 2017). Executive function skills are the general-purpose mechanisms that regulate cognition and action, including working memory, inhibition, and cognitive flexibility, and are largely regulated by prefrontal cortical brain areas (Miyake & Friedman, 2012). Attention supports goal-directed behaviors such as alerting and orienting to stimuli and maintaining engagement with, or switching between, stimuli (Petersen & Posner, 2012). Attention supports the development and use of executive function skills, and these cognitive skills have overlapping and interdependent developmental time courses with one another and with speech and language skills (Shokrkon & Nicoladis, 2022). Furthermore, executive function and attention skills exhibit rapid development during the preschool-age period, the time during which stuttering onset occurs for most children. Inefficient or ineffective regulation of executive function and attention skills may contribute to dysregulated or inefficient planning and execution of speech production, resulting in disfluent speech (Anderson & Ofoe, 2019; Chang et al., 2018).

Previous studies have identified subtle differences in working memory, especially phonological working memory, inhibition, cognitive flexibility, and attention in children who stutter compared with children who do not stutter. However, findings are inconsistent across studies and may reflect other factors, including child age, task and methodology, and other speech and language abilities, such as phonological skills (Anderson & Ofoe, 2019; Ofoe et al., 2018; Paphiti & Eggers, 2022; Paphiti et al., 2022; Rocha et al., 2019). Together, prior research suggests that inefficient executive function and attention skills may impact the formulation and execution of speech in individuals who stutter, especially in more complex situations. However, additional studies with larger participant groups and more systematic evaluation of these cognitive skills are needed.

#### Interim discussion: Research priorities relevant to cognitive factors in stuttering

- Systematic evaluation of executive function and attention skills in children who stutter compared with children who do not stutter across simple and complex tasks that tap into single and multiple cognitive skills simultaneously is needed.
- We can benefit from studies of the development of executive function and attention skills in children who stutter from early preschool through adolescence.
- Because these skill areas are known to be highly inter-related, we should continue to explore interactions of executive function and attention skills with language formulation and speech production skills in children who stutter compared with children who do not stutter.

#### Social-cognitive features of stuttering events

Social cognition plays a critical role in triggering or at least shaping overt stuttering events, thus contributing to the hallmark variability of stuttering. For example, many adults who stutter report that they do not stutter during private speech, or overt speech intended only for the speaker (Jackson et al., 2021). This suggests that stutterers’ perceptions of a listener, whether real or imagined, are critical and necessary for stuttering to occur. As suggested by Alm (2014), the speech motor system in people who stutter may be susceptible to interference from (normal levels of) social cognition. Stutterers also learn to anticipate stuttering events, or sense that upcoming speech will be stuttered if that speech is executed as initially planned (Jackson et al., 2015). Anticipation is pervasive, such that most if not all stutterers, school age or older, anticipate the occurrence of their own stuttered speech to some degree (Jackson et al., 2015, 2018, 2019). Stutterers respond to anticipation, prior to speech execution, by altering their speech plan (stalling, word switching) and sometimes bypassing a stuttering event completely (e.g., choosing not to talk because they will stutter; Jackson et al., 2015, 2018, 2019; Rodgers & Jackson, 2021). Thus, anticipation has a direct impact on stuttering events because it can change how they are presented and whether they manifest overtly at all. Given the mostly covert nature of anticipation, brain imaging is a useful method to search for neural markers that precede overtly stuttered speech. For example, recent work points to activity in the right dorsolateral prefrontal cortex as a possible correlate of stuttering anticipation in adults (Jackson et al., 2022). It may be that these patterns reflect a form of *proactive* inhibitory control, a prospective inhibitory mechanism that slows or delays an upcoming (aversive) event, whereas other work points to possible *reactive* inhibitory control indexed by right presupplementary motor area activity (Orpella et al., 2024). Future work should develop methods to uncover the role of social-cognitive factors in triggering or contributing to overtly stuttered speech.

#### Interim discussion: Research priorities relevant to social-cognitive factors in stuttering

- It will be useful to test whether children close to the onset of stuttering stutter during private as well as typical conversational speech. This will help determine whether social-cognitive factors are critical and necessary for stuttering to occur, and not just contributors that develop with experience.
- We can benefit from research to determine neural substrates of social and cognitive processes preceding speech execution and their impacts.
- Because of its relevance to therapeutic intervention, research should determine when anticipation begins to develop during the early years of stuttering in children—when this awareness enters consciousness and when it starts impacting speech planning and execution.

### Clinical Management Approaches in Stuttering

#### Defining aims for stuttering intervention

As with other current re-evaluations of the potential focus on neurotypicality in clinical populations, contemporary discussions about stuttering intervention consider the premise that stuttered speech does not, in itself, need to be viewed as disordered (Campbell et al., 2019; Constantino, 2022; Gerlach-Houck & Constantino, 2022). Though it is widely recognized that stuttering arises from an underlying pathology reflected in neural differences, evolving perspectives recognize that *all* people speak with different levels of fluency, just as they show varying levels of other skills. Some people speak more fluently, and some people speak less fluently, and this diversity reflects the reality of the human condition (Campbell et al., 2019; Reeves et al., 2023; Reeves & Yaruss, 2024). This anti-ableist view of stuttering does not negate the importance of intervention for those who want it; it does, however, highlight ways that clinicians and researchers can account for the perspectives of people who stutter who do not view themselves as disordered when planning intervention. In particular, considering the goals of people who stutter necessitates an understanding of the experience of stuttering beyond the observable speech characteristics that have historically been the focus of so much research and treatment (see, e.g., Connery et al., 2020; Isaacs & Swartz, 2021; Quesal, 2010; Tichenor & Yaruss, 2019a, 2019b; Yaruss & Quesal, 2004).

Certainly, many people want to speak more fluently (Venkatagiri, 2009); thus, research into appropriate methods for addressing disfluent speech remains a priority. As noted above, however, existing treatment methods are largely inadequate for meeting the goals of people who stutter beyond the early preschool period. Many adult stutterers report shortcomings of fluency-focused therapies (Tichenor & Yaruss, 2019a; Yaruss et al., 2002) and highlight the greater need to reduce the *impact* of stuttering on their lives (Beilby et al., 2013; Boyce et al., 2022; Briley, Ellis, & Jacobs, 2021; Craig et al., 2009; Daniels & Gabel, 2004; Klein & Hood, 2004; Klompas & Ross, 2004; Tichenor, Palasik, & Yaruss, 2023; Tichenor & Yaruss, 2020, 2021). Studying the views of people who stutter regarding the broader implications of living with stuttering yields valuable opportunities to ensure that what is important to stutterers—the life impacts of stuttering—remains important to clinicians and researchers. Similar approaches have been taken in the field of aphasia research, yielding a host of novel treatment approaches that incorporate all aspects of life impacted by communication disorders (Chapey et al., 2000). Taking a comprehensive view of stuttering ensures that any effective intervention aligns with the goals of people who stutter and yields meaningful improvements in their lives.

#### Interim discussion: Research priorities relevant to defining aims for stuttering intervention

- It would be valuable to better understand mechanisms associated with the underlying “loss of control” as experienced by people who stutter, rather than focusing primarily on apparent severity of disfluency as defined by listeners (e.g., clinicians and researchers). This includes specifying what the loss of control actually is (neurological underpinnings), what factors precipitate it (e.g., linguistic, paralinguistic), and why it varies across tasks, times, and settings.
- Better understanding of the ways in which adverse impact associated with stuttering develops from onset over the lifespan is needed so that appropriate interventions can be developed to minimize or even prevent adverse impact. This research should include improved understanding of personal characteristics of individuals who stutter (both innate and learned), as well as contributions from society (e.g., stigma and discrimination), that combine to cause negative experiences for people who stutter that might be amenable to targeted interventions.

#### The impact of stuttering: Relevance to goals and therapy approaches

The term *adverse impact* as it relates to stuttering refers to the negative personal reactions (e.g., negative thoughts, feelings, and behaviors) and the broader speech- or communication-related limitations experienced by a person who stutters (Tichenor & Yaruss, 2019b; Yaruss & Quesal, 2004, 2006, 2016). Though many researchers have highlighted the various forms that adverse impact may take, the field has been limited in its understanding and treatment of adverse impact by the continued use of surface severity (i.e., the frequency or duration of overt stuttered events) as the primary measure used to (a) screen subjects for participation in research, (b) interpret research data, and (c) ascertain treatment outcomes. Surface severity is often measured at single time points and in tasks that are low in ecological validity compared to real-world communication, despite the fact that stuttering behaviors are highly variable (Constantino, 2012; Tichenor & Yaruss, 2021; Yaruss, 1997). Surface severity is also only weakly associated with other forms of adverse impact, including cognitive-affective reactions and broader life consequences (Blumgart et al., 2012; Ward et al., 2021). Further, adolescents and adults who are more fluency focused in their goals when speaking actually experience significantly higher levels of adverse impact than those who aim to allow themselves to stutter openly (Tichenor, Gerwin, & Walsh, 2023; Tichenor, Palasik, & Yaruss, 2023; Tichenor, Walsh, et al., 2022), suggesting that the pursuit or desire of fluency may not prevent adverse impact but contribute to it (see Constantino et al., 2020, and Tichenor, Constantino, & Yaruss, 2022, for discussion of spontaneous vs. controlled fluency). Thus, there is a critical need for a broader view of stuttering severity beyond counts of observable stuttered events; such research could better reflect the ways in which subgroups of people who stutter experience, cope with, and live with stuttering *differently* from other people who stutter and lead to improved therapeutic approaches and outcomes.

#### Interim discussion: Research priorities relevant to setting goals for stuttering therapy

- We need a better understanding of heterogeneous profiles that present in the clinic. Much stuttering research over decades has used population-level indicators to guide therapeutic decisions. For example, research has shown that, as a group, adults who stutter are at increased risk for generalized anxiety, social anxiety, depression, and suicidal ideation compared to adults who do not stutter (Briley, Gerlach, & Jacobs, 2021; Craig & Tran, 2014). Although these findings are meaningful in understanding the group or population-level risk of these adverse outcomes, they cannot identify an individual client’s risk given the heterogeneity of how stutterers experience, cope with, and manage stuttering in their lives.
- We need to develop effective clinical practices that integrate both population and individual perspectives—knowing who a person is in terms of how they developed and were shaped in their environment (population perspective) and who they are in terms of their life choices and desires (individual or subgroup perspective; see Arah, 2009, for discussion). There is a critical need to study individual or subgroup perspectives to better understand the underlying mechanisms of stuttering over the lifespan from onset through development and into adulthood that can inform improved clinical management.

#### Issues in treating stuttering in very young children

The primary goal of most therapy for very young children who stutter is amelioration of stuttering behaviors (Guitar, 2014; Manning & DiLollo, 2017); it is primarily indirect in nature (modifying the child’s speaking environment, rather than using direct instruction of the child), employs numerous component strategies, and is typically administered by parents. Although well-designed trials of therapy outcomes are fairly rare, available evidence suggests little difference between treatment and unassisted recovery, both of which hover around 70% (Bloodstein et al., 2021). In addition, mechanisms of action specifically tied to improvements in fluency, when observed, are poorly understood (Bernstein Ratner, 2005). Indeed, past and current work casts significant doubt on many conventional childhood stuttering therapy components, such as behavioral contingencies for fluent speech (Lidcombe therapy; Donaghy et al., 2015; Swift et al., 2016), changes in parent conversational style (Garbarino & Bernstein Ratner, 2022) or alterations to the pacing and content of parent–child interactions (Burns & Bernstein Ratner, 2022; Godsey & Bernstein Ratner, 2025) that aim to reduce communicative pressure on the child. Asking parents to administer therapies with unknown effectiveness risks delaying implementation of therapies that might reduce adverse impact of stuttering in children who persist, while elevating parental guilt if recovery is not seen. In addition, controversies over which existing therapies are preferable to others (de Sonneville-Koedoot et al., 2015; Nippold, 2018; O’Brian et al., 2023) may have stultified efforts either to improve the existing multifaceted therapies by further investigating mechanisms of action or to develop newer, innovative approaches. Finally, it is unclear why most therapies for children at the onset of stuttering continue to define success as elimination of symptoms, rather than provision of self-efficacy and management strategies (for the children and their parents; Shenker et al., 2023), more in keeping with other health conditions that arise during the toddler and preschool years, such as diabetes, rheumatoid arthritis, or asthma (Lozano & Houtrow, 2018).

#### Interim discussion: Research priorities relevant to treatment in children who stutter

- We need cooperative, large-scale clinical trials of existing therapies, with short-term waitlist controls and multiyear follow-up to compare therapy outcomes with historical data on unassisted recovery from stuttering.
- More studies (both laboratory-based and observational) that seek to detangle the effectiveness of individual therapy components within complex, multi-faceted interventions are needed.
- We should encourage researchers to expand the scope of successful therapy outcomes for young children to include assessment that goes beyond speech fluency measures.

## ADDITIONAL RESEARCH PRIORITIES RAISED DURING BREAKOUT SESSIONS

Both interim and culminating breakout sessions were held in five groups each comprising seven to eight attendees. Discussions were facilitated via prompts that asked each group to generate a list of priority areas for future research in stuttering. All attendees convened after each breakout session to report on each group’s discussion points and reconvened for a final set of full group discussions. The research priorities reported in this article are those that emerged as consensus opinions following final group discussion following 2 days of topically organized research presentations and ensuing discussion. Some continued earlier themes, while other priorities reflected relative absence of work perceived to be critical for next stages of understanding the nature of stuttering and clinical approaches to its management. Wording reflected here was refined by the original topic presenters and the two workshop co-chairs.

### Etiology, Mechanisms, and Novel Treatment Development

In the last 2 decades, a growing body of literature has contributed to significant advances in our understanding of possible mechanisms of stuttering. However, many outstanding questions remain about the etiological and biological bases of the disorder, which has hampered progress in developing efficacious treatments that may target core deficits underlying stuttering. The attendees identified key challenges and priority areas for research necessary to achieve breakthroughs in this area.

#### Large-scale consortium studies, clinical trials, international collaborations

Research in the area of stuttering has often led to inconclusive and inconsistent findings, due in large part to small-N studies with insufficient sample sizes conducted by research groups working in silos. As shown in neighboring disciplines that have formed large research consortia for genetics and neuroimaging research (e.g., in the fields of ADHD, autism, and schizophrenia), progress, new discoveries, and breakthroughs in health-related disciplines can be expedited through consortium research that convenes multiple research groups, enabling studies with sufficient power to generate rigorous and replicable findings. Consortia can be formed around basic research questions as well as clinical aims, including clinical trials that may span international investigators and sites.

#### Increasing diverse subject sampling in research that better represents the stuttering population

For any clinical research project, it is important to sample participants who adequately represent the population under study. Challenges to diverse sampling within the field of stuttering have been discussed in prior papers (Falk et al., 2013; Green et al., 2022) and bias in study participant sampling is expected to have occurred in many studies in stuttering to date. Sources of potential bias may have included but are not limited to conducting research in major university settings (which limit potential participants varying in socioeconomic and other demographic factors), solely recruiting from clinics rather than from the wider community, and excluding participants with co-occurring conditions such as ADHD and other neurodevelopmental conditions, to name a few; each of these limitations threatens interpretations of prior findings. Bias in sampling limits the generalizability of the research findings and can lead to inconsistent, nonreplicable results across different studies. During the discussion, additional relevant questions were raised, including whether people who stutter and their families are included in the formulation and conduct of scientific research questions, and who the scientific questions are designed to help and how. In particular, the importance of including stutterers themselves during research planning and interpretation of findings was raised, so that study questions, design, and implementation can be guided by fundamental questions that matter to those who are affected.

#### Defining subtypes of stuttering: Neural phenotyping and subtyping to better understand heterogeneity, and development of targeted individual-specific therapeutic interventions

The outward manifestations of stuttering comprise a well-recognized set of behaviors (e.g., sound–syllable repetitions, prolongations, and blocks, sometimes accompanied by physical concomitants such as tensing of facial muscles or extremity movement) that set stuttering apart as a distinct and readily recognizable condition from other speech motor disorders. However, there is great heterogeneity among speakers in terms of the severity of symptoms (e.g., frequency and duration of stuttered moments), exacerbating factors (such as comorbid disorders or weaknesses), emotional reactivity/resilience, and perceived impact of stuttering on quality of life (Tichenor & Yaruss, 2019a, 2021), to name only a few factors that distinguish individuals who stutter from a generic profile. Diagnostic and intervention studies, as well as basic science studies, have all proposed the presence of subgroups within the stuttering population (Ajdacic-Gross et al., 2018; Alm & Risberg, 2007; Ambrose et al., 2015; Blood & Seider, 1981; Foundas et al., 2004; Frigerio-Domingues et al., 2019; Riley & Riley, 1980; Schwenk et al., 2007; Seery et al., 2007; Yairi, 2007). Drug responsiveness to pharmaceutical interventions for stuttering also varies across speakers who stutter (Gordon et al., 1995; Stager et al., 2005), further suggesting the existence of subgroups within the population. There is an increasing interest in neural phenotyping to discover potential neuroanatomical and neurophysiological subtypes among speakers who stutter, which may then help target different intervention approaches for specific subtypes. Finding neural subtypes in particular may enable precision medicine or neurostimulation approaches in stuttering intervention that lead to better treatment outcomes in the future.

#### Investigating the neurophysiological bases of “loss of control” in ecologically valid contexts

Though stuttering occurs in the context of social communication, few experimental research studies have implemented testing or observational conditions that are ecologically valid for stuttering. For example, the vast majority of neuroimaging studies has been conducted in clinical settings or in scanner environments that induce less stuttering than might be representative of the speaker’s usual speech. Interacting with clinicians (rather than unfamiliar listeners), loud scanner environments, such as in fMRI, and producing single words without communicative intent all lead to relatively fluent speech production in people who stutter in typical research environments. Furthermore, most studies exclude from analysis instances of stuttering that occur during the experiment due to concomitant noise issues (e.g., excessive movement, emotional reactivity, compensatory behaviors) associated with the moments of stuttering; these instances are furthermore too infrequent to examine with sufficient statistical power. Brain imaging analyses have focused on relationships to overt stuttered utterances rather than mental and/or emotional states (e.g., the experience of loss of control that is commonly reported by speakers who stutter). The brain responses associated with stuttering have been suggested to be compensatory responses to such loss of control. Then, what is loss of control neurophysiologically? The challenge for future researchers would be to tackle this question by conducting studies in social settings and in contexts where speech occurs with communicative intent, and by examining brain responses before, during, and after the stuttered event, while controlling for the confounding factors associated with the stuttering moment.

#### Novel treatment development informed by advances in neurobiological understanding of stuttering

A greater understanding of etiology can drive better treatment development. Several approaches were brought up during presentations and discussions that target modulating brain function and chemistry. In terms of *neuromodulation*, initial promising effects on enhancing speech fluency with tDCS in stuttering speakers were presented, summarized in Neural Bases of Stuttering in Adults, above. In addition to continuing with more research with transcranial electrical stimulation methods applied in conjunction with fluency inducing conditions, some raised the need to further explore the potential of TMS and DBS methods as neuromodulation interventions to treat stuttering. The importance of conducting theory-driven work to guide better neural targeting for such brain stimulation treatments was also discussed. Others also asked whether more research should be done in the area of pharmacology/psychopharmacology.

### Shifting Perspectives on Setting Treatment Goals and What Constitutes Successful Outcomes in Stuttering Intervention

The workshop highlighted a diversity of perspectives regarding the objectives of therapeutic interventions for stuttering, which should, in theory, relate to both to basic science as well as clinical trials. On one hand, the need for the development of novel treatments that target changes in the fluency of speech for many individuals seeking improved fluency was discussed as important. On the other hand, a growing consensus in the field emphasized the importance of going beyond a restricted focus on changing the outward speech symptoms associated with stuttering. These ideas were expressed by many attendees and discussed extensively by the speakers who presented on this topic (see Clinical Management Approaches in Stuttering, above). Breakout session discussants raised additional ideas that warrant future investigation in this area.

#### Defining outcomes for treatment

A theme that emerged during the conference was the critical issue of defining desired outcomes of treatments for stuttering. This is not necessarily as straightforward as might be imagined in other conditions impacting health and well-being. There was a general agreement that stuttering intervention should seek to enhance spontaneity, authenticity, and increased communicative function and participation on an individual level. Conventional treatment approaches that focus only on reducing instances of stuttering were criticized as leading to unnatural-sounding and effortful speech production that is difficult for a speaker to maintain (thus leading to relapse). Moreover, such a focus can lead to potentially negative impact on self-perceptions, and in some cases even contribute to an increased frequency of stuttering following treatment (Yaruss et al., 2002). The importance of making the primary goal of stuttering therapy effective communication rather than fluency was voiced by many, while most participants agreed that all therapy goals should be individual specific. In all cases, speakers and discussants agreed that desired therapy outcomes should prioritize *reducing negative impact on the lives of those who stutter across the lifespan, from childhood through adulthood*.

#### Training and education of clinicians

Beyond improvements in basic and clinical knowledge, substantial concern was voiced about a need to study implementation science in stuttering treatment. In particular, given concerns raised about existing therapies, how do we break the cycle of focusing only on fluency by speech-language pathologists? (Recent articles have discussed the problem of changing existing, well-entrenched therapy approaches as “de-implementation science,” which seems apt in this discipline; Walsh-Bailey et al., 2021). This should include providing options to families and speakers who seek treatments that primarily focus on fluency—ideally moving beyond stating, “I don’t want to/want my child to stutter,” as the sole intervention goal. In both existing practice and in the training of future clinicians, many voiced the need to explore when and how to have these conversations, and how to help speech-language pathologists themselves expand their understanding and acceptance of stuttering.

As with many other conditions, some raised the need to explore how to change the understanding and acceptance of nonfluency in society and the environment more broadly as we work to build a society that is more accepting of differently abled individuals. Additionally, a questionable track record of effective therapies that focus narrowly on speech fluency may explain why surveys suggest that clinicians are not comfortable working with people who stutter. Many voiced the opinion that the field would need to make progress in identifying factors that determine best treatment outcomes, given multiple possible therapy objectives. Finally, there was a strong consensus that there is a need to explore potential differences in therapy objectives for adults and children who stutter and how heterogeneity in individual profiles may impact response to treatment.

## CONCLUSIONS

Research findings over the past few decades have contributed to a better understanding of the nature of stuttering, a speech disorder once considered a medical mystery. Now widely considered a complex neurodevelopmental condition, many questions nevertheless remain about its potential biological bases; furthermore, a mechanistic explanation linking biology to behavior remains lacking. These gaps in our knowledge have hindered systematic efforts in developing science-guided novel interventions—a pressing need for those who seek treatment to improve their ability to communicate freely, effortlessly, and authentically. In this article summarizing a recent NIDCD sponsored workshop focused on stuttering research, project leaders summarize updated scientific findings and guided discussion of future priorities for basic and clinical research that could pave the way for breakthroughs in the field. Large-scale, multidisciplinary collaborations that support and strengthen rigorous basic science and clinical trials, increasing diversity of sampling, and broadening our perspectives on targets for stuttering intervention, emerged as some major topics proposed to overcome past barriers to progress. While these themes represent a broad sampling of research priorities discussed at the meeting, we acknowledge that additional emerging themes and approaches could also bear potential to advance stuttering research. Pursuing and successfully achieving these research priorities have the potential to make meaningful impacts toward enhancing effective communication and reducing negative life consequences experienced by many children and adults who stutter.

## ACKNOWLEDGMENTS

NIDCD provided funding and staff support to convene the virtual workshop that generated the ideas reported in this article. The authors thank Yolanda L. Jones, National Institutes of Health Library Editing Service, for editing assistance.

## FUNDING INFORMATION

Jennifer Below, National Institute on Deafness and Other Communication Disorders (https://dx.doi.org/10.13039/100000055), Award ID: R01DC017175. Jennifer Below, National Institute on Deafness and Other Communication Disorders (https://dx.doi.org/10.13039/100000055), Award ID: R03DC015329. Soo-Eun Chang, National Institute on Deafness and Other Communication Disorders (https://dx.doi.org/10.13039/100000055), Award ID: R01DC011277. Soo-Eun Chang, National Institute on Deafness and Other Communication Disorders (https://dx.doi.org/10.13039/100000055), Award ID: R01DC018283. Ho Ming Chow, National Institute on Deafness and Other Communication Disorders (https://dx.doi.org/10.13039/100000055), Award ID: R21DC015853. Frank Guenther, National Institute on Deafness and Other Communication Disorders (https://dx.doi.org/10.13039/100000055), Award ID: R01DC007683. Nan Bernstein Ratner, National Institute on Deafness and Other Communication Disorders (https://dx.doi.org/10.13039/100000055), Award ID: R01DC015494. Nan Bernstein Ratner, National Institute on Deafness and Other Communication Disorders (https://dx.doi.org/10.13039/100000055), Award ID: R01DC307764. Bridget Walsh, National Institute on Deafness and Other Communication Disorders (https://dx.doi.org/10.13039/100000055), Award ID: R03DC013402. Bridget Walsh, National Institute on Deafness and Other Communication Disorders (https://dx.doi.org/10.13039/100000055), Award ID: R01DC018000. Ludo Max, National Institute on Deafness and Other Communication Disorders (https://dx.doi.org/10.13039/100000055), Award ID: R01DC017444. Ludo Max, National Institute on Deafness and Other Communication Disorders (https://dx.doi.org/10.13039/100000055), Award ID: R01DC020707. Amanda Hampton Wray, National Institute on Deafness and Other Communication Disorders (https://dx.doi.org/10.13039/100000055), Award ID: R21DC017227. Amanda Hampton Wray, National Institute on Deafness and Other Communication Disorders (https://dx.doi.org/10.13039/100000055), Award ID: R01DC019904. Shahriar SheikhBahaei, National Institute of Neurological Disorders and Stroke (https://dx.doi.org/10.13039/100000065), Award ID: ZIA NS009420. Nan Bernstein Ratner, National Stuttering Association, Award ID: CASE grant. Nan Bernstein Ratner, National Science Foundation (https://dx.doi.org/10.13039/100000001), Award ID: BCS1626300. Kate Watkins, Medical Research Council (https://dx.doi.org/10.13039/501100000265), Award ID: MR/K023772/1 and MR/N025539/1. Kate Watkins, NIHR Oxford Biomedical Research Centre (https://dx.doi.org/10.13039/501100013373), Award ID: NIHR203316. Kate Watkins, Wellcome Trust (https://dx.doi.org/10.13039/100010269), Award ID: 203139/Z/16/Z and 203139/A/16/Z. National Institute on Deafness and Other Communication Disorders (https://dx.doi.org/10.13039/100000055), Award ID: R01DC00559.

## AUTHOR CONTRIBUTIONS

**Soo-Eun Chang**: Conceptualization; Methodology; Writing – original draft; Writing – review & editing. **Jennifer E. Below**: Conceptualization; Writing – original draft; Writing – review & editing. **Ho Ming Chow**: Conceptualization; Writing – original draft; Writing – review & editing. **Frank H. Guenther**: Conceptualization; Writing – original draft; Writing – review & editing. **Amanda M. Hampton Wray**: Conceptualization; Writing – original draft; Writing – review & editing. **Eric S. Jackson**: Conceptualization; Writing – original draft; Writing – review & editing. **Ludo Max**: Conceptualization; Writing – original draft; Writing – review & editing. **Nicole E. Neef**: Conceptualization; Writing – original draft; Writing – review & editing. **Shahriar SheikhBahaei**: Conceptualization; Writing – original draft; Writing – review & editing. **Lana Shekim**: Conceptualization; Writing – review & editing. **Seth E. Tichenor**: Conceptualization; Writing – original draft; Writing – review & editing. **Bridget Walsh**: Conceptualization; Writing – original draft; Writing – review & editing. **Kate E. Watkins**: Conceptualization; Writing – original draft; Writing – review & editing. **J. Scott Yaruss**: Conceptualization; Writing – original draft; Writing – review & editing. **Nan Bernstein Ratner**: Conceptualization; Methodology; Writing – original draft; Writing – review & editing.

## Supplementary Material



## TECHNICAL TERMS

*Gene expression:*: The transformation of the information encoded in a gene into a functional form, such as a protein or RNA molecule.
Phenotypes: Traits that can be observed and are the product of interactions between genes and the environment.
Genome-wide association studies (GWAS): A research approach that aims to identify specific genomic variants statistically linked to the risk of developing a particular disease or trait.
Genomic variant: Refers to a lasting alteration in the DNA sequence of a gene.
Genotype: Refers to the genetic composition of a cell or organism, which plays a role in determining its phenotype.
Animal model: A non-human species used in biomedical research to replicate certain aspects of biological processes or diseases found in humans.
Connectome: Refers to a comprehensive map of neural connections in the brain, which is often visualized as a “wiring diagram”.
Neuromodulation: A treatment method that achieves therapeutic effects by modifying the function of the nervous system and its electrophysiologic signals.
Transcranial electrical stimulation (tES): tES, such as transcranial direct current stimulation (tDCS) and transcranial alternating current stimulation (tACS), is a noninvasive brain stimulation technique used to alter brain function.
