# Supplementary material for: Stuttering: Our Current Knowledge, Research Opportunities, and Ways to Address Critical Gaps
Source: Neurobiol Lang (Camb). 2025 Apr 2;6:nol_a_00162. doi: 10.1162/nol_a_00162 (PMC11977836; doi:10.1162/nol_a_00162)
Supplement: Supplementary file 1 [file nol-6-1-162-s001.pdf]

List of speakers and corresponding sections that summarized their presentations in this article

2.1 Biological bases of stuttering

Progress in understanding the genetics of stuttering: Jennifer Below, The Vanderbilt Genetics Institute, Vanderbilt University Medical Center, Nashville, TN, USA

Animal models: Shahriar SheikhBahaei, Neuron-Glia Signaling and Circuits Unit, National Institute of Neurological Disorders and Stroke (NINDS), National Institutes of Health (NIH), Bethesda, MD, USA

2.2 Demographic, physiological, and neurological bases of stuttering persistence and recovery in childhood

Demographic and physiological factors: Bridget Walsh, Department of Communicative Sciences and Disorders, Michigan State University, East Lansing, MI, USA

Neuroanatomical factors: Soo-Eun Chang, Department of Psychiatry, Michigan Medicine, University of Michigan, Ann Arbor, MI, USA; Department of Communication Disorders, Ewha Womans University, Seoul, Korea

Neurophysiological factors: Ho Ming Chow, Department of Communication Sciences and Disorders, University of Delaware, Newark, DE, USA; Bridget Walsh, Department of Communicative Sciences and Disorders, Michigan State University, East Lansing, MI, USA

2.3 Neural bases of stuttering in adults

Variations in brain structure and function among adults who continue to experience stuttering/ Impact of stuttering interventions on adult brain function: Nicole Neef, Department of Diagnostic and Interventional Neuroradiology, University Medical Center Göttingen, Georg August University, Göttingen, Germany

New interventions that seek to alter brain connectivity to enhance fluent speech: Kate Watkins, Department of Experimental Psychology, University of Oxford, Oxford, UK

2.4 Understanding speech motor control in stuttering

Auditory-motor integration: Ludo Max, Department of Speech & Hearing Sciences, University of Washington, Seattle, WA, USA

Computational modeling of the speech motor control system: Frank H. Guenther, Departments of Speech, Language, & Hearing Sciences and Biomedical Engineering, Boston University, Boston, MA, USA

2.5 Cognitive and social aspects of stuttering

Executive function and attention in developmental stuttering: Amanda Hampton Wray, Department of Communication Science and Disorders, University of Pittsburgh, Pittsburgh, PA, USA

Social-cognitive features of stuttering events: Eric Jackson, Department of Communicative Sciences and Disorders, New York University, New York, NY, USA

2.6 Clinical management approaches in stuttering

Defining aims for stuttering intervention: Scott Yaruss, Department of Communicative Sciences and Disorders, Michigan State University, East Lansing, MI, USA

The impact of stuttering: relevance to goals and therapy approaches: Seth Tichenor, Department of Speech-Language Pathology, Duquesne University, Pittsburgh, PA, USA

Issues in treating stuttering in very young children: Nan Bernstein Ratner, Department of Hearing and Speech Sciences & Program in Neuroscience and Cognitive Science, University of Maryland, College Park, MD, USA
